# Draft Genome Sequence of *Robinsoniella peoriensis* 6600698, a Confounder of *Clostridium difficile* Diagnosis

**DOI:** 10.1128/genomeA.01275-16

**Published:** 2016-11-17

**Authors:** Chrissy h. Roberts, Helen Alexandra Shaw, Natalie Ferguson, Mark Holland, Brendan W. Wren, Richard A. Stabler

**Affiliations:** a Faculty of Infectious and Tropical Diseases, London School of Hygiene and Tropical Medicine, London, United Kingdom; b Microbiology, Maidstone Hospital, Maidstone, Kent, United Kingdom

## Abstract

*Robinsoniella peoriensis* is a Gram-positive, strictly anaerobic, spore-forming, rod-shaped organism. Here, we report the draft genome of *R. peoriensis* 6600698, initially classified as *Clostridium difficile* due to growth on selective agar, a fecal *gdh* PCR-positive result, and clinical symptoms. *R. peoriensis* is a potential confounder of *C. difficile* diagnosis.

## GENOME ANNOUNCEMENT

*Robinsoniella peoriensis* was first identified from swine manure and manure storage pits (1) as a Gram-positive, strictly anaerobic, spore-forming, rod-shaped organism. Contemporaneously, an isolate from a deep wound on the heel of a 79-year-old person was submitted to the University of Gothenburg Culture Collection (1). Subsequently, there have been reports of clinical disease linked to *R. peoriensis* (2–4). *R. peoriensis* has been isolated from a presumptive *Clostridium difficile* diarrhea sample that tested negative for *C. difficile* toxins (5), and commensal *R. peoriensis* has been isolated from healthy, premature human neonate twins (6).

*R. peoriensis* 6600698 was originally isolated from an 82-year-old male on ChromID (bioMérieux) *C. difficile* selective agar plates, incubated anaerobically. The patient had elevated levels of fecal lactoferrin (IBD Scan, Techlab, Blacksburg, VA, USA), indicating intestinal inflammation. The bacteria were initially classified as nontoxigenic *C. difficile* due to clinical symptoms, a positive *C. difficile* glutamate dehydrogenase (GDH) (C. DIFF CHEK, TechLab), and negative toxigenic culture (Premier Toxins A&B, Meridian Bioscience, Cincinnati, OH, USA). *C. difficile* PCR ribotyping (7) produced a “sporadic” or uncommon/new PCR ribotype that did not match existing standards. Attempts to identify the multilocus sequence type (MLST) profile (8) failed to amplify a single allele; therefore, whole-genome sequencing was performed.

*R. peoriensis* 6600698 was sequenced using an Illumina MiSeq (2 × 250 bp) and an Oxford Nanopore Technologies MinION MKI nanosequencer (Oxford Nanopore Technologies, United Kingdom) (ENA accession no. PRJEB15237). Sequence data were analyzed using Metrichor Agent version 2.40.17. The Illumina MiSeq generated 3,500,296 reads and 602 Mb from two runs. The MinION generated 6,245 reads and 22 Mb (maximum read length, 34 kb; median, 3,083 bp; *N*_50_, 5,171 bp) from a single run. MiSeq reads were polished using Trimmomatic version 0.33 (9). MinION reads were extracted into FASTA format using poretools version 0.5.1 (10). A draft genome was assembled using SPAdes version 3.6.2 (11). The assembled contigs were further polished using GapFiller version 1.10 (12) and Pilon version 1.16 (13). Contigs were annotated using Prokka version 1.11 (14). The draft genome consisted of 160 contigs, totaling 7,202,111 bp with 41% G+C; 61 tRNAs, 5,745 coding sequences, and 2 clustered regularly interspaced short palindromic repeats were also present. A BLAST analysis of the assembled 16S ribosomal sequence showed a 1,522/1,524 bp (>99%) identity with *R. peoriensis* PPC44 (AF445283), and the top seven matches were all *R. peoriensis* (29 February 2016).

*R. peoriensis* 6600698 contains a putative glutamate dehydrogenase (*gdh*) (gene 6600698_02412) that shares low-level homology to the *C. difficile* R20291 *gdh* (137/408 identities; 204/408 positives; bit score, 190; E-value, 1 × 10^−55^), which may explain the false *gdh* PCR-positive result. No *C. difficile* pathogenicity locus toxin genes or gene remnants were present in the pathogenicity locus. *R. peoriensis* and *C. difficile* are both members of the order *Clostridiales* and common features of both can result in this emerging human pathogen being misdiagnosed as nontoxigenic *C. difficile*.

### Accession number(s).

This whole-genome shotgun project has been deposited at EMBL under the accession numbers FMJR01000001 to FMJR01000160. The version described in this paper is the first version.
